# PUBLIC HEALTH POLICY IN AN AGING POPULATION

**DOI:** 10.1093/geroni/igac059.1504

**Published:** 2022-12-20

**Authors:** Eileen Flores, Danielle Llaneza, Hanamori Skoblow, Lilian Azer, Patricia D’Antonio

**Affiliations:** Penn State, State College, Pennsylvania, United States; Rutgers University, Piscataway, New Jersey, United States; University of Missouri, Columbia, Missouri, United States; University of California, Riverside, Riverside, California, United States; The Gerontological Society of America, Washington, District of Columbia, United States

## Abstract

In the U.S., cancer and its related issues have an impact on public health, such as increased death rates and economic burdens due to lost productivity and associated treatment costs. Along the cancer control continuum, there are ample opportunities to enact policies that can improve cancer prevention, detection, and survivorship. As the US population ages, health policies that address barriers to cancer prevention and optimal survivorship care for older adults will be key. The intersection of policy and public health has gained momentum in the last decade. Changes resulting from the Affordable Care Act and President Biden’s recently set goals (i.e. reduce age-adjusted death rates from cancer by at least 50% and improving the experience of people and their families living with and surviving cancer) for the Cancer Moonshot program have ignited the role of public health policy and its implications among the US population. As a policy intern, I had the opportunity to advocate on behalf of GSA, the nation’s largest aging organization, for research initiatives (e.g. ARPA-H) that amplify opportunities to address policies in public health. Advocating for initiatives that accelerate the government’s biomedical and health research to embrace a geroscience approach will continue to spearhead much needed opportunities to directly impact policies surrounding aging and cancer.

